# Gel-Like Human Mimicking Phantoms: Realization Procedure, Dielectric Characterization and Experimental Validations on Microwave Wearable Body Sensors

**DOI:** 10.3390/bios11040111

**Published:** 2021-04-08

**Authors:** Sandra Costanzo, Vincenzo Cioffi, Adil Masoud Qureshi, Antonio Borgia

**Affiliations:** 1 Dipartimento di Ingegneria Informatica, Modellistica, Elettronica e Sistemistica, Università della Calabria, 87036 Rende, Italy; v.cioffi@dimes.unical.it (V.C.); adil.qureshi@unical.it (A.M.Q.); a.borgia@dimes.unical.it (A.B.); 2 National Research Council of Italy (CNR), Institute for Electromagnetic Sensing of the Environment (IREA), 80124 Naples, Italy; 3 ICEmB (Inter-University National Research Center on Interactions between Electromagnetic Fields and Biosystems), 16145 Genoa, Italy; 4 CNIT (Consorzio Nazionale Interuniversitario per le Telecomunicazioni), 43124 Parma, Italy

**Keywords:** biosensors, biological phantoms, wearable devices, security

## Abstract

A simple and low-cost procedure for gel-like time-durable biological phantoms is presented in this work. Easily accessible materials are adopted, which are able to provide a flexible and controllable method to rapidly realize different kind of tissues. The proposed technique is applied to fabricate various tissue-mimicking phantoms, namely skin, muscle, blood and fat. Their effectiveness is first tested by performing dielectric characterization on a wide frequency range, from 500 MHz up to 5 GHz, and validating the measured dielectric parameters (dielectric constant and conductivity) by comparison with reference models in the literature. Then, a multi-layer phantom simulating the human arm is realized, and a wearable body sensor is adopted to prove the perfect agreement of the biometric response achieved in the presence of the fabricated phantom and that provided by a real human arm.

## 1. Introduction

We live in an increasingly connected world, where communication devices far outnumber the human inhabitants [1]. Most people in the evolved world own multiple portable or wearable devices at all times, in the form of smartphones, laptops, smartwatches, health and fitness trackers. In order to ensure safe and reliable operation in the biomedical context [2], the interaction of the above devices with the human body should be carefully analyzed and understood. Ideally, new devices should be designed and tested on actual users, to ensure their performances are coherent with the expected design. However, apart from being unsafe and unethical, using an actual human as a test subject also presents a host of logistical problems. The bulk of design and development is actually performed by adopting computer-aided simulations, in order to save time and reduce costs [3]. Prototyping at each design stage for validation with a real human body would dramatically increase costs and slow down the product development cycle. Furthermore, even at the later stages of development, it is not always convenient or possible to use a human subject. Human beings find it hard to stay completely stationary for extended durations, reducing repeatability of sensitive tests. For tests conducted in specialized chambers, accommodating an actual user would require a larger enclosure that would be costlier [4]. Thus, in all but the rarest of cases, designers rely on human body phantoms and surrogates which mimic the human body. As may be expected, the human body is a very complex system, and it is not feasible to build an exact replica that is able to duplicate all of its properties. Therefore, most phantoms are limited to the scope of a specific biomedical application. Various phantoms and dummies have been developed and discussed in the literature that replicate breast tissue [5], dry phantoms for SAR testing [6], optical properties of skin [7], etc.

In the case of handheld and wearable telemedicine devices, designers are often interested in the interaction between onboard antennas and the human body [8]. Since the major part of the body is a high permittivity lossy medium [9], it can have significant effects on the radiation pattern and efficiency of antennas. The position of a wearable device on the body impacts the types of underlying body tissue that it will be exposed to. For example, a device placed on the arm is exposed to a different set of tissues as compared to a device placed on the chest or head. Fortunately, the high conductivity of the external tissue layers [10] reduces the penetration depth, thus limiting the interaction of internal organs with antennas. Therefore, for the purposes of antenna design and validation, it may be sufficient to model just the external layers, including skin, blood, fat and muscle [11,12]. The electrical properties of these layers have been studied at length, and empirical data as well as numerical models are widely available [13,14]. The known properties of these layers are commonly used to create simplistic multi-layer phantoms in 3D electromagnetic simulations. A planar model offers significant savings in computation time and resources, as compared to more complex models. However, the validity of such non-homogeneous planar models has not been rigorously discussed in the literature.

The current work describes the first results (to the best of the authors’ knowledge) of the realization and validation of a multi-layer phantom, that reproduces dielectric characteristics widely quoted in the literature. Cheap and easily available ingredients are used for creating each layer of the gelatinous phantom. Different electrical characteristics are achieved by simply changing the ratio of ingredients. A rigorous experimental validation of the complex dielectric permittivity for the realized samples is carried out. The work also compares the ability of the realized multi-layer phantom to accurately mimic the behavior of the human body. To this end, a wearable antenna usable for biometric security purposes is adopted as a test device, by comparing its sensing performances on a voluntary person as well as on the human-mimicking phantom, along with results obtained using 3D EM simulations.

## 2. Materials and Methods

In this section, the procedure adopted to realize a multi-layer phantom, usable for the design and the pre-clinical test of telemedicine sensors, is described. Low-cost and easily available ingredients are adopted, so to have a simple and cheap realization process. Specifically, food-grade gelatine, in particular gelatine leaves, are used as the solidifying agent. The composition originally proposed by [15] is refined to achieve a better agreement with theoretical models. It is observed that higher oil content corresponds to lower permittivity phantoms, while a higher water content results in a higher overall permittivity. The conductivity of the phantom is directly influenced by the salt concentration. Dishwashing soap is used as an emulsifying agent that serves to combine the water and oil into an emulsion. In Table 1, the proportions of the different ingredients adopted in this paper for the phantom’s realization are reported. The updated composition leads to a better reproduction of the dielectric characteristics of the human body.

### 2.1. Phantom Realization Procedure

The gelatine leaves are soaked in water for about 10 min, and they are subsequently squeezed to remove excess water. The rehydrated gelatine is then heated up to about 80 °C, until it is completely liquified. The liquid gelatine is then cooled down to around 45 °C, before the remaining ingredients are added. It is important to continuously stir the mixture during this process in order to avoid the formation of clumps. The blend is then heated again to obtain a more homogenous mixture, which is then poured into an 11 cm by 8.4 cm container, and stored in a fridge overnight.

Four different phantoms, namely fat, skin, blood and muscle, are realized by changing the percentage of the involved ingredients. Details of the involved quantities for the different human tissues are provided in Table 1.

### 2.2. Measurement Setup for Experimental Validations

A specific measurement setup is considered to perform the accurate experimental validation of the biological phantom, which is realized through two sequential steps, namely:

1. The dielectric characterization of each phantom layer;
2. The performance characterization of the multi-layer phantom on a prototype of wearable antenna working in contact with the human body.

The experimental setup is equipped in the Microwave Laboratory at the University of Calabria; it includes an open-ended coaxial probe (type DAK-3.5 built by SPEAG [16]), which is connected to Vector Network Analyzer (VNA) Anritsu, model MS 4647A. The above setup is able to measure the complex dielectric permittivity (dielectric constant and tangent loss) in the range from 1 to 200, within a frequency interval going from 200 MHz up to 20 GHz. It introduces a measurement uncertainty equal to ±2%, as reported in the Calibration Certificate provided by the Calibration Laboratory of SPEAG.

The fields radiated by the open-ended probe interact with the material in contact with its interface. The VNA is able to measure the magnitude and the phase of the reflected signal, which is directly related to the dielectric properties of the contact material. To achieve accurate results, the Medium Under Test (MUT), namely the biological phantom in this specific case, must appear as a semi-infinite layer, i.e., it must have a greater extension than the diameter of the sensor opening. Furthermore, it is important that no air gap exists between the sensor and the sample. Due to the gel-like nature of the sample, the above conditions are easily guaranteed, as a good contact can be obtained between the probe and the MUT, by applying a little pressure on to the probe. 

As a first step, the above instruments are calibrated using a distilled water sample at room temperature, specifically equal to 23 °C at the time of measurement. After the calibration phase, the complex permittivity of the reference materials is measured to gauge the instrument accuracy. Table 2 presents the percentage variance observed in the measured complex permittivity of the reference materials (deionized water and air) after the calibration. Repeated measurements were conducted for different frequencies, it can be seen that all the measurements are extremely close to the expected value. 

## 3. Experimental Results

In this section, the experimental results assessing the reliability of the proposed tissue-mimicking phantoms are presented. Specifically, in the first subsection, the measurement results relative to the complex permittivity (both dielectric constant as well as conductivity) of the realized phantoms are reported and validated through reference models; while, in the second subsection, the experimental validation of the multi-layer phantom simulating the human arm is performed by verifying the proper response of a wearable sensor designed to operate for communication in direct contact with a human person.

### 3.1. Dielectric Characterization of Realized Phantoms

**Figure 1:**
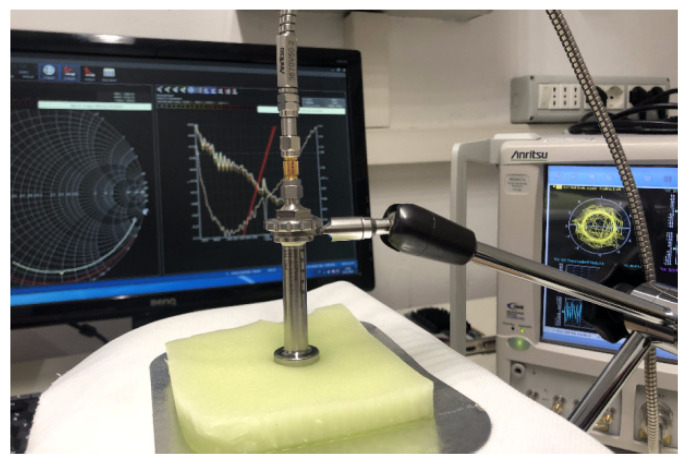
Test setup in the Microwave Laboratory at University of Calabria.

Following the procedure described in Section 2.1, dielectric measurements are performed by adopting the test setup illustrated in Figure 1.

**Figure 2:**
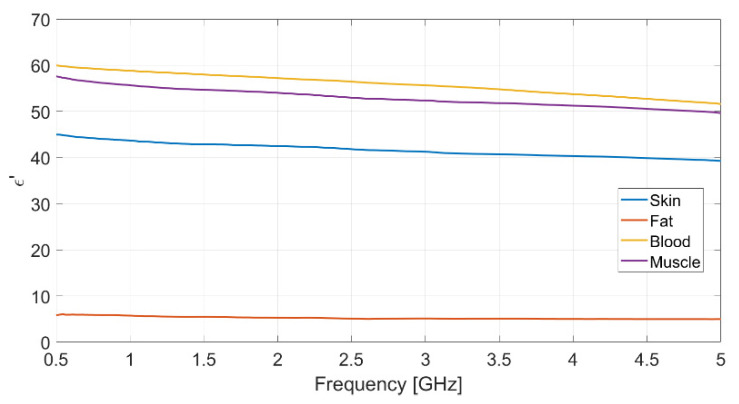
Measured real part of the relative permittivity vs. frequency for each realized bio-phantom.

**Figure 3:**
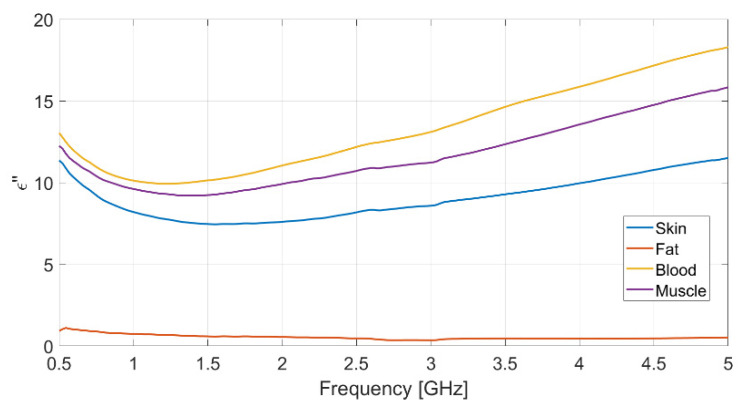
Measured imaginary part of the relative permittivity vs. frequency for each realized bio-phantom.

**Figure 4:**
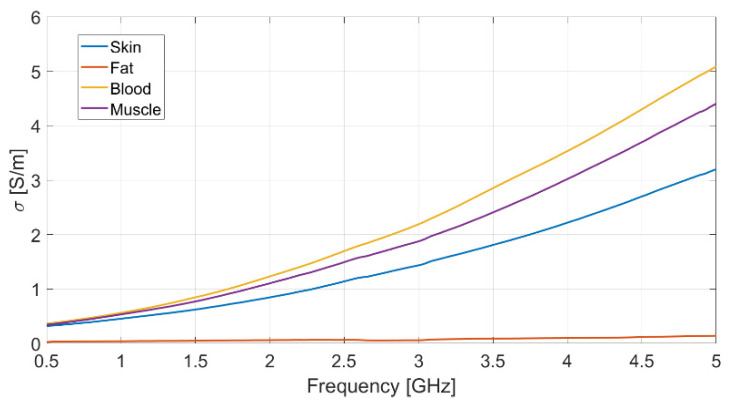
Measured conductivity vs. frequency for each realized bio-phantom.

Each measurement was repeated ten times, to ensure data reliability and measurement repeatability, and the mean value for each set of measurements was assumed. The resulting measured data for dielectric permittivity (real and imaginary part) as well as conductivity of each biological phantom are reported in Figure 2, Figure 3 and Figure 4, respectively.

The dielectric characterization was performed over a wide frequency range, from 500 MHz up to 5 GHz, so as to construct a big database including the dielectric parameters of the bio-phantoms, to be successfully adopted for a large variety of applications, including biosensors, bio-imaging and tissue engineering. The aforementioned frequency range covers most of the frequency bands used for common biomedical and diagnostic applications as well as off-body and on-body communication.

The measured values for the dielectric parameters are successfully validated by comparison with data taken from reference models in the literature [14,17]. As a demonstration, the above comparison is reported in Table 3 for the 2.4 GHz frequency, operating within the Industrial, Scientific, Medical (ISM) band. It is assumed as the working frequency for the wearable body sensor described in the following subsection.

### 3.2. Wearable Body Sensor for Tissue Engineering Application

The final validation presented in this subsection is aimed at demonstrating the close mimicking features of the realized biological phantoms, as compared to real body tissues.

Specifically, a multi-layer phantom is first realized by following the experimental procedure described in Section 2; then, a prototype of miniaturized wearable sensor realized by the research group at University of Calabria [18], and operating within the ISM frequency band (2.4–2.5 GHz), is adopted as a test body sensor to validate its functionality when working in the presence of a human body (simulated by the multi-layer phantom).

**Figure 5:**
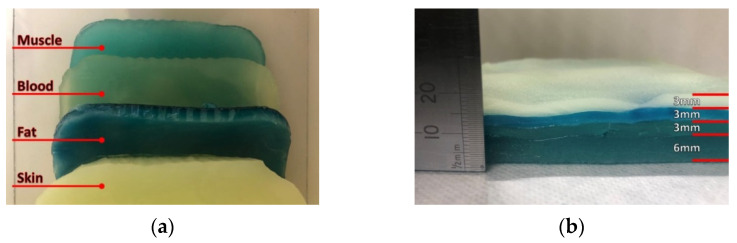
Realized multi-layer biological phantom: (**a**) top view and (**b**) side view.

In order to realize the multi-layer phantom, thin slices are cut from the realized samples of the various body layers (skin, blood, muscle, fat), which are subsequently stacked and then placed on a polycarbonate sheet (Figure 5), with the aim to provide a mechanical support as well as an easy handling during testing.

**Figure 6:**
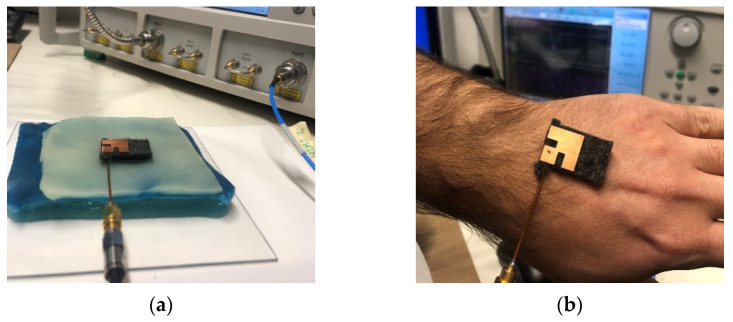
Setup for return loss measurement with (**a**) multi-layer phantom and (**b**) human body.

**Figure 7:**
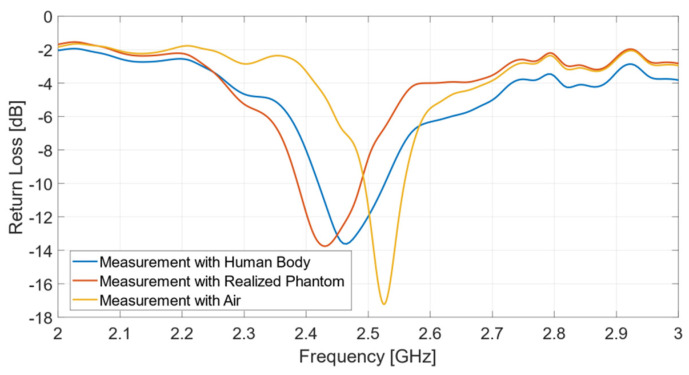
Return loss measurements: comparison between multi-layer phantom and human body.

Once its fabrication was complete, the effectiveness of the multi-layer phantom was tested by measuring the reflection response (return loss) of the wearable body sensor placed on it (Figure 6a). The result is successfully compared (Figure 7) with that obtained from measurements directly performed on a voluntary person (Figure 6b).

It can be observed that the antenna exhibits a resonant frequency equal to 2.43 GHz, when placed on the multilayer phantom, and 2.465 GHz, if operating on the human body. The usable impedance bandwidth is equal to 102 MHz (4.2%) with the phantom, and 105 MHz (4.26%) in the presence of a real human body. On the other hand, the measurement in air exhibits a resonance frequency at 2.53 GHz and an impedance bandwidth of 64 MHz (2.5%). The measured curves reported in Figure 7 reveal the excellent performance of the biological multi-layer phantom, whose response is almost identical to that provided by a real human arm, thus opening to a variety of useful biomedicine applications, such as biosensors, where pre-clinical tests can be successfully implemented by adopting a proper tissue-mimicking phantom, thus avoiding direct measurements on human persons.

## 4. Discussion

A simple and low-cost procedure, adopting easily accessible materials, has been presented for the realization of gel-like human phantoms that excellently mimick the behavior of real human tissues.

Liquid phantoms are the most commonly adopted in the scientific community, as they are the easiest and most flexible to fabricate, with volume and consistency being easily controllable. Solid phantoms may be used for lifetime extension, but they require more complicated and less flexible procedures. Gelatin phantoms can provide semi-solid solutions, requiring less realization time, while preserving a relatively long temporal stability. Moreover, they can be successfully adopted for imaging and biocompatibility applications.

Following the above motivations, we have successfully implemented an easy technique to realize gel-like tissue-mimicking phantoms. First, the fabrication procedure has been accurately described, and particularized for the following tissues: skin, muscle, blood and fat. Successively, the dielectric characterization of the fabricated biological phantoms has been performed, and parameters (dielectric permittivity and conductivity) from the experimental stage have been successfully validated with existing models in the literature, thus confirming the accurate behavior of the realized tissue phantoms. As a subsequent step, a multi-layer phantom mimicking the human arm has been realized by properly sizing and stacking the single prototyped biological layer. Finally, a wearable body sensor useful for biometric security applications has been adopted to demonstrate the perfect agreement between the sensor response provided in the presence of the fabricated multi-layer phantom and that obtained with a real human arm.

The achieved results present the reliable application of the proposed gel-like human phantoms as tissue-mimicking materials for in vitro studies and prediction of in vivo bioeffects at microwave and millimeter-wave frequencies.

## Figures and Tables

**Table 1:** Materials concentration for the different tissue-mimicking phantoms.

Phantom Type	Water		Gelatine		Oil		Soap		Salt	
	[g]	%	[g]	%	[g]	%	[g]	%	[g]	%
Fat	58	14	15	3.6	330	80	10	2.4	-	-
Skin	34	55	6	10	19	30	1.8	2.8	1.4	2.2
Blood	153	70	23	10	15	7	27	12	1.2	1
Muscle	100	30	18	6	200	60	10	4	-	-

**Table 2:** Percentage variance in the complex permittivity measurement of reference materials at different frequencies.

Frequency	Air ε′	Air ε″	Water ε′	Water ε″
500 MHz	0.0064	0.1193	0.2760	0.3142
1 GHz	0.1757	0.0098	0.0355	0.0164
2 GHz	0.0150	6.16 × 10^−10^	0.1629	0.1564
3 GHz	0.0029	3.89 × 10^−6^	0.0774	0.0368
4 GHz	0.0023	2.67 × 10^−5^	0.1154	0.0190
5 GHz	1.39 × 10^−4^	2.78 × 10^−4^	0.0343	0.2264

**Table 3:** Measured and reference dielectric parameters at 2.4 GHz.

Phantom Type	Measured ε′	Reference ε′	Difference	Measured σ	Reference σ	Difference
Fat	5.43	4.93	10%	0.06	0.09	33%
Skin	42.58	42.93	0.8%	1.07	1.55	31%
Blood	57.01	58.36	2.3%	1.59	2.49	36%
Muscle	53.61	52.34	2.4%	1.40	1.31	7%

## Data Availability

The data presented in this study are available on request from the corresponding author.
